# Prevalence and Risk Factors of Chronic Kidney Disease among Type 2 Diabetes Patients: A Cross-Sectional Study in Primary Care Practice

**DOI:** 10.1038/s41598-020-63443-4

**Published:** 2020-04-10

**Authors:** Janjira Jitraknatee, Chidchanok Ruengorn, Surapon Nochaiwong

**Affiliations:** 1 Kidney Center, Sansai Hospital, Chiang Mai, 50290 Thailand; 2 Department of Pharmaceutical Care, Faculty of Pharmacy, Chiang Mai University, Chiang Mai, 50200 Thailand; 3 Pharmacoepidemiology and Statistics Research Center (PESRC), Faculty of Pharmacy, Chiang Mai University, Chiang Mai, 50200 Thailand

**Keywords:** Diabetes complications, Kidney diseases, Chronic kidney disease

## Abstract

This cross-sectional study aimed to investigate the prevalence and risk factors of chronic kidney disease (CKD) among 1,096 primary care type 2 diabetes (T2DM) patients in northern Thailand between October 2016 and September 2017. CKD was defined as estimated glomerular rate filtration values of <60 mL/min/1.73 m^2^. Prevalence with confidence intervals across CKD advanced stages 3–5 were estimated. Factors associated with CKD were evaluated by multivariate logistic regression. The overall prevalence of CKD was 24.4% (21.9–27.0), with severities of 11.4% (9.7–13.4), 6.8% (5.5–8.5), 4.6% (3.5–6.0), and 1.6% (1.0–2.5) for stages 3 A, 3B, 4, and 5, respectively. Regarding age and glycaemic control, individuals older than 75 years and those with a haemoglobin A1c ≥ 8% had the highest prevalence of 61.3% (51.7–70.1) and 38.6% (34.3–43.2), respectively. The multivariable logistic regression model explained 87.3% of the probability of CKD. The six independent significant risk factors of CKD were older age, retinopathy, albuminuria, haemoglobin A1c ≥ 7%, anaemia, and uric acid>7.5 mg/dL. A relatively high prevalence of CKD, especially in older patients and those with diabetic complications-related to poor glycaemic control, was encountered in this primary care practice. Early identification may help to target optimise care and prevention programs for CKD among T2DM patients.

## Introduction

Globally, the total number of people with diabetes is estimated to increase from 415 million (8.8%) in 2015 to 642 million (10.4%) in 2040, with the largest alterations expected to occur in the urban population of low- to middle-income countries (LMICs)^1^. Of them, type 2 diabetes mellitus (T2DM) accounts for more than 90% of people with diabetes^2,3^. By 2040, the difference worldwide is projected to broaden, with 477.9 million affected people living in urban areas and 163.9 million in rural areas^1^. It has been postulated that the burden of diabetes and its complications in the LMICs may be contributed by the economic development and rapid urbanisation via increased caloric intake and the adoption of a sedentary lifestyle^2,4,5^. More importantly, the most striking demographic change to diabetes prevalence in global terms also seems to be related to the growth of the proportion of the elderly population^6^.

Despite rates of diabetes-related complications such as cardiovascular disease decreasing significantly in the past two decades, it has not translated nearly as well as kidney complications^7^. Approximately 10% of deaths in people with T2DM are attributable to kidney failure^8^. It is well-established that diabetes-related chronic kidney disease (CKD) is the leading cause of end-stage kidney disease (ESKD) in T2DM patients worldwide^9,10^. In the United States, 2013–2016, approximately 36% of patients with diabetes develop diabetic kidney disease resulting in persistent albuminuria, a reduced estimated glomerular filtration rate (eGFR), or both^11^. Interestingly, the risk of diabetes-related CKD is observed much higher in Asian countries than in Western countries^12^. Moreover, diabetes patients in developing countries are at a particularly increased risk of developing kidney complications compared to those in developed countries^13^. As the global burden of diabetes increases dramatically due to T2DM^1,14,15^, the annual growth rate of diabetes-related CKD is expected to rise as well, particularly in LMICs.

Based on the available evidence from LMICs, there is considerable heterogeneity of CKD among urban and rural areas^16^. Moreover, the aetiology of CKD among T2DM patients in LMICs are multifactorial and affected by the burden of both non-communicable and communicable diseases compared with high-income countries. In more urbanised areas, unhealthy lifestyles—a high-fat diet and physical inactivity—may accelerate the higher prevalence of diabetes and its complications. In a nationwide survey, the prevalence of CKD (eGFR <60 mL/min/1.73 m^2^) was 35.4% in Thai T2DM patients^17^. However, wide variations in the prevalence of CKD among T2DM patients were observed across geographical regions and residential areas in Thailand. For instance, the prevalence of CKD among T2DM patients in urbanised areas (capital city and its vicinities) was 37.2%^18^, whereas 24.3–25.3% were observed for those who lived in less urbanised communities or regional areas^19,20^. To date, data on the epidemiology of CKD among T2DM patients in the suburban area are limited, which may indicate differences in urban and rural areas.

As renal replacement therapy (RRT) for ESKD treatment is not always available to CKD patients in LMICs owing to the limit of healthcare resources^21^, the routine surveillance for and identification of T2DM patients who are at high risk of CKD is urgently needed to decrease healthcare burden and costs. It is also critical to identify risk factors of diabetes-related CKD for its prevention, detection, and treatment to alleviate the rising burden of ESKD worldwide. To address this knowledge gap, this cross-sectional study investigated the prevalence and risk factors of CKD among T2DM patients in a primary care setting within a suburban community in northern Thailand.

## Results

### Characteristics of T2DM patients

A total of 1,368 T2DM patients were screened between October 1, 2016 and September 30, 2017. Of them, 274 were excluded (Supplementary Fig. S1). Consequently, 1,094 patients were included in the analysis. Patient characteristics are illustrated in Table 1. Of all values, 0.0–1.7% were missing for the cohort. Most patients were female (57.1%), with a mean age ± standard deviation (SD) and median diabetes duration ± interquartile range (IQR) of 61.6 ± 11.1 years and 5.9 ± 10.1 years, respectively. The mean systolic and diastolic blood pressures of the study populations were 132.4 ± 18.2 and 77.6 ± 11.1, respectively. Among antihypertensive medications, angiotensin-converting enzyme inhibitors or angiotensin II receptor blockers were most commonly prescribed (59.0%). Oral antidiabetic drugs with metformin monotherapy (21.8%) or metformin plus sulfonylurea (34.7%) were the most common treatments for glycaemic control.

**Table 1:** Patient characteristics according to CKD status.

Characteristic	All Patients (n = 1,094)	Missing Data	With CKD (n = 267)	Without CKD (n = 827)	*P* value
**Socio-demographic**					
Male	469 (42.9)	0 (0.0)	115 (43.1)	354 (42.8)	0.943
Age, year	61.6 ± 11.1	0 (0.0)	68.0 ± 10.1	59.6 ± 10.6	<0.001
BMI, kg/m^2^	24.8 ± 4.9		24.4 ± 4.8	25.0 ± 4.9	0.124
Smoking status					
Never	967 (88.4)	0 (0.0)	239 (89.5)	728 (88.0)	0.575
Former	37 (3.4)		10 (3.8)	27 (3.3)	
Current	90 (8.2)		18 (6.7)	72 (8.7)	
Alcohol consumption					
Never	887 (81.1)	0 (0.0)	228 (85.4)	659 (79.7)	0.109
Former	43 (3.9)		9 (3.4)	34 (4.1)	
Current	164 (15.0)		30 (11.2)	134 (16.2)	
Insurance status					
UCS by NHSO	819 (74.9)	0 (0.0)	205 (76.8)	614 (74.2)	0.553
CSMBS	139 (12.7)		34 (12.7)	105 (12.7)	
SSS/others	136 (12.4)		28 (10.5)	108 (13.1)	
Medical history					
Hypertension	890 (81.4)	0 (0.0)	239 (89.5)	651 (78.7)	<0.001
CAD	88 (8.0)	0 (0.0)	41 (15.4)	47 (5.7)	<0.001
CBVD	79 (7.2)	0 (0.0)	34 (12.7)	45 (5.4)	<0.001
Any grade retinopathy	153 (14.0)	0 (0.0)	75 (28.1)	78 (9.4)	<0.001
Albuminuria	579 (52.9)	0 (0.0)	187 (70.0)	392 (47.4)	<0.001
Systolic BP, mmHg	132.4 ± 18.2	0 (0.0)	135.7 ± 22.2	131.4 ± 16.6	0.001
Diastolic BP, mmHg	77.6 ± 11.1	0 (0.0)	75.7 ± 12.3	78.2 ± 10.6	0.001
Duration of diabetes, year (median ± IQR)	5.9 ± 10.1	0 (0.0)	8.0 ± 13.9	5.1 ± 8.8	<0.001
**Laboratory values**					
Serum creatinine, mg/dL (median ± IQR)	0.9 ± 0.4	0 (0.0)	1.4 ± 0.6	0.8 ± 0.3	<0.001
eGFR mL/min per 1.73 m^2^	78.5 ± 26.7	0 (0.0)	40.6 ± 14.1	90.7 ± 16.3	<0.001
Fasting plasma glucose, mg/dL	155.1 ± 60.8	0 (0.0)	160.6 ± 76.7	153.4 ± 54.7	0.092
Haemoglobin A1c, %	8.0 ± 1.9	0 (0.0)	8.5 ± 1.9	7.8 ± 1.8	<0.001
Haemoglobin, g/dL	12.6 ± 2.0	0 (0.0)	11.6 ± 2.1	12.9 ± 1.9	<0.001
Uric acid, mg/dL	6.1 ± 1.5	33 (3.0)	7.2 ± 1.6	5.7 ± 1.3	<0.001
Total cholesterol, mg/dL	195.2 ± 48.2	19 (1.7)	195.5 ± 52.2	195.0 ± 47.0	0.897
Triglycerides, mg/dL (median ± IQR)	140 ± 104	18 (1.6)	144 ± 93	137 ± 106	0.363
LDL-C, mg/dL	116.7 ± 41.2	19 (1.7)	118.3 ± 42.6	116.1 ± 40.8	0.460
HDL-C, mg/dL	46.9 ± 13.0	19 (1.7)	45.3 ± 13.7	47.4 ± 12.8	0.020
Non-HDL-C, mg/dL	148.2 ± 45.3	19 (1.7)	150.2 ± 48.5	147.6 ± 44.2	0.420
**Glycaemic control**					
Diet only	109 (9.9)	0 (0.0)	72 (8.7)	37 (13.9)	<0.001
Metformin only	238 (21.8)		201 (24.3)	37 (13.9)	
Sulfonylurea only^†^	138 (12.6)		73 (8.8)	65 (24.3)	
Metformin plus sulfonylurea	380 (34.7)		326 (39.4)	54 (20.2)	
Other oral antidiabetic drugs^‡^	3 (0.3)		1 (0.1)	2 (0.8)	
Insulin only	85 (7.8)		42 (5.1)	43 (16.1)	
Insulin plus metformin	51 (4.7)		45 (5.4)	6 (2.2)	
Insulin plus sulfonylurea	29 (2.6)		16 (1.9)	13 (4.9)	
Insulin plus metformin plus sulfonylurea	61 (5.6)		51 (6.2)	10 (3.8)	
**Other medication use**					
ACEIs/ARBs	645 (59.0)	0 (0.0)	151 (56.6)	494 (59.7)	0.391
Beta-blockers	153 (14.0)	0 (0.0)	64 (24.0)	89 (10.8)	<0.001
CCBs	401 (36.6)	0 (0.0)	105 (39.3)	296 (35.8)	0.307
Loop diuretic	82 (7.5)	0 (0.0)	51 (19.1)	31 (3.8)	<0.001
Thiazide	74 (6.8)	0 (0.0)	18 (6.7)	56 (6.8)	1.000
Other antihypertensive agents^§^	55 (5.0)	0 (0.0)	27 (10.1)	28 (3.4)	<0.001
No. of antihypertensive therapy, (median ± IQR)	1 ± 1	0 (0.0)	2 ± 1	1 ± 1	<0.001
Statins	657 (60.0)	0 (0.0)	167 (62.6)	490 (59.2)	0.351
Fibrates	83 (7.6)	0 (0.0)	20 (7.5)	63 (7.6)	1.000
Antiplatelet agents	521 (47.6)	0 (0.0)	146 (54.7)	375 (45.3)	0.009
Allopurinol	16 (1.5)	0 (0.0)	7 (2.6)	9 (1.1)	0.081
Colchicine	25 (2.3)	0 (0.0)	12 (4.5)	13 (1.6)	0.009

Values are numbers with percentages in parentheses or expressed as mean ± SD, unless otherwise indicated.

^†^Includes glibenclamide, glipizide.

^‡^Includes pioglitazone only, metformin plus pioglitazone, metformin plus glipizide plus pioglitazone.

^§^Includes hydralazine, methydopa, doxazosin.

Abbreviations: ACEIs/ARBs, angiotensin-converting enzyme inhibitors/angiotensin II receptor blockers; BMI, body mass index; BP, blood pressure; CAD, coronary artery disease; CBVD, cerebrovascular disease; CCBs, calcium channel blockers; CKD, chronic kidney disease; CSMBS, Civil Servant Medical Benefit Scheme; eGFR, estimated glomerular filtration rate; HDL-C, high density lipoprotein cholesterol; IQR, interquartile range; LDL-C, low density lipoprotein cholesterol; NHSO, National Health Security Office; SSS, Social Security Scheme; UCS, Universal Coverage Scheme.

### Prevalence of CKD among T2DM patients

**Figure 1:**
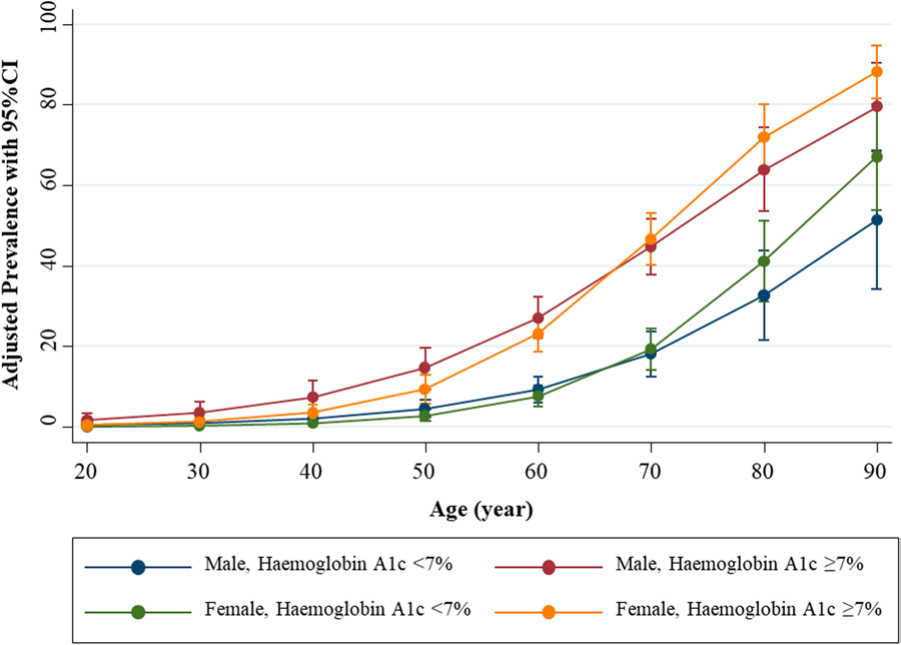
Age, sex and glycaemic control adjusted prevalence rates of CKD in patients with T2DM. Abbreviations: CI, confidence interval; CKD, chronic kidney disease; HbA1c, haemoglobin A1c; T2DM, type 2 diabetes mellitus.

The estimated mean eGFR by the CKD Epidemiology Collaboration (CKD-EPI) equation for T2DM patients was 78.5 ± 26.7 mL/min/1.73 m^2^. The overall unadjusted prevalence of CKD (eGFR <60 mL/min/1.73 m^2^) was 24.4% (95% confidence interval [CI], 21.9–27.0; Table 2). Age, sex, and glycaemic control adjusted prevalence rates of CKD in patients with T2DM are illustrated in Fig. 1. With respect to CKD stage, the unadjusted prevalence of stage 3 A (eGFR, 45–59 mL/min/1.73 m^2^), stage 3B (eGFR, 30–44 mL/min/1.73 m^2^), stage 4 (eGFR, 15–29 mL/min/1.73 m^2^), and stage 5 (eGFR, <15 mL/min/1.73 m^2^) were 11.4% (95% CI, 9.7–13.4), 6.8% (95% CI, 5.5–8.5), 4.6% (95% CI, 3.5–6.0), and 1.6% (95% CI, 1.0–2.5), respectively (Table 2).

**Table 2:** Age-, sex- and glycaemic control-specific prevalence of CKD in patients with T2DM.

Characteristics	N	Stage 3A		Stage 3B		Stage 4		Stage 5		Overall CKD	
		Cases	Prevalence Estimated (95% CI)	Cases	Prevalence Estimated (95% CI)	Cases	Prevalence Estimated (95% CI)	Cases	Prevalence Estimated (95% CI)	Cases	Prevalence Estimated (95% CI)
**Age, year**											
<45	68	1	1.5 (0.2–9.8)	1	1.5 (0.2–9.8)	1	1.5 (0.2–9.8)	0	0.0	3	4.4 (1.4–12.9)
45–55	219	15	6.8 (0.4–11.1)	5	2.3 (1.0–5.4)	1	0.4 (0.1–3.2)	3	1.4 (0.4–4.2)	24	11.0 (7.4–15.8)
56–65	439	38	8.6 (6.4–11.7)	26	5.9 (4.1–8.6)	19	4.3 (2.8–6.7)	8	1.8 (0.9–3.6)	91	20.7 (17.2–24.8)
66–75	262	37	14.1 (10.4–18.9)	24	9.2 (6.2–13.3)	18	6.9 (4.4–10.6)	5	1.9 (0.8–4.5)	84	32.1 (26.7–38.0)
>75	106	34	32.1 (23.8–41.6)	19	17.9 (11.7–26.4)	11	10.4 (5.8–17.8)	1	0.9 (0.1–6.4)	65	61.3 (51.7–70.1)
**Sex**											
Male	469	56	11.9 (9.3–15.2)	34	7.2 (5.2–10.0)	19	4.0 (2.6–6.3)	6	1.3 (0.6–2.8)	115	24.5 (20.8–28.6)
Female	625	69	11.0 (8.8–13.8)	41	6.6 (4.9–8.8)	31	5.0 (3.5–7.0)	11	1.8 (1.0–3.2)	152	24.3 (21.1–27.8)
**Glycaemic control, haemoglobin A1c (%)**											
<6	103	9	8.7 (4.6–16.0)	6	5.8 (2.6–12.4)	5	4.8 (2.0–11.2)	2	1.9 (0.5–7.5)	22	21.4 (14.5–30.4)
6–6.9	292	22	7.5 (5.0–11.2)	7	2.4 (1.1–5.0)	2	0.7 (0.2–2.7)	3	1.0 (0.3–3.1)	34	11.6 (8.4–15.9)
7–7.9	241	17	7.0 (4.4–11.1)	8	3.3 (1.7–6.5)	8	3.3 (1.7–6.5)	1	0.4 (0.1–2.9)	34	14.1 (10.2–19.1)
≥8	458	77	16.8 (13.6–20.5)	54	11.8 (9.1–15.1)	35	7.6 (5.5–10.5)	11	2.4 (1.3–4.3)	177	38.6 (34.3–43.2)
**All**	**1,094**	**125**	**11.4 (9.7–13.4)**	**75**	**6.8 (5.5–8.5)**	**50**	**4.6 (3.5–6.0)**	**17**	**1.6 (1.0–2.5)**	**267**	**24.4 (21.9–27.0)**

Abbreviations: CI, confidence interval; CKD. chronic kidney disease; T2DM, type 2 diabetes mellitus.

Notably, a high prevalence across CKD stages in elderly T2DM patients (>65 years) was observed, particularly in individuals older than 75 years (61.3%; 95% CI, 51.7–70.1 for the overall unadjusted prevalence of CKD). Similarly, there was a high prevalence across CKD stages among patients with poor glycaemic control, particularly in individuals with a haemoglobin A1c ≥ 8% (24.4%; 95% CI, 21.9–27.0 for the overall unadjusted prevalence of CKD). Nonetheless, there was no substantial difference in the sex-specific prevalence of CKD. Of these, the unadjusted CKD prevalence according to sex-specific was 24.5% (95% CI, 20.8–28.6) and 24.3% (95% CI, 21.1–27.8) in male and female, respectively (Table 2, Fig. 1).

### Risk factors of CKD among T2DM patients

With regard to patient characteristics, the univariate logistic regression recognised 21 candidate risk factors with *P*-values less than 0.100, including age, body mass index (BMI), alcohol consumption, hypertension, coronary artery disease, cerebrovascular disease, retinopathy, albuminuria, systolic blood pressure, long-standing diabetes (>10 years), haemoglobin A1c, haemoglobin, uric acid, glycaemic control, medication usage (beta-blockers, loop diuretics, other antihypertensive agents, antiplatelet agents, allopurinol, and colchicine), and number of antihypertensive therapy (Supplementary Table S1).

**Figure 2:**
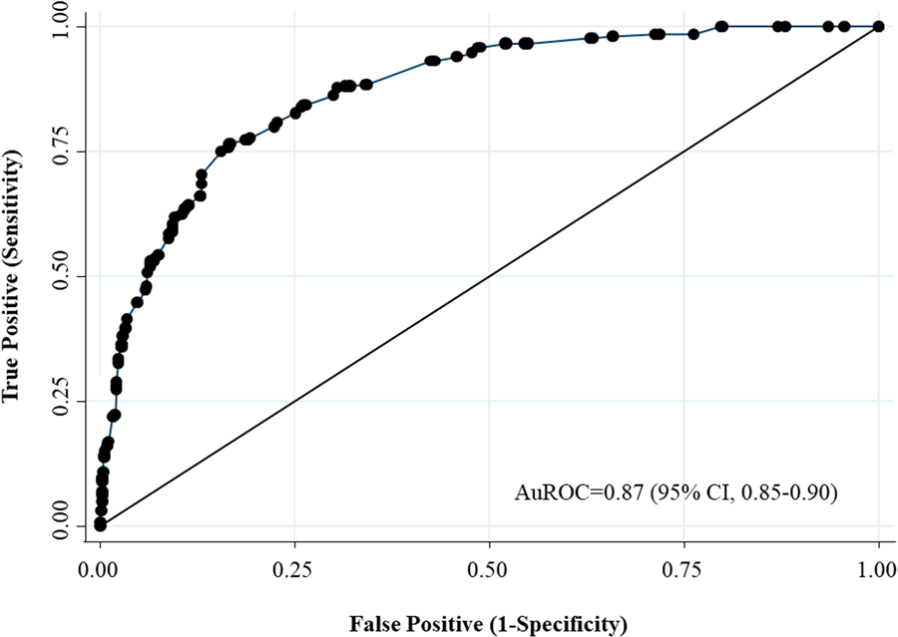
The AuROC curve and 95%CI of the risk factors of CKD in patients with T2DM. Abbreviations: AuROC, area under the receiver operating characteristic; CI, confidence interval; CKD, chronic kidney disease; T2DM, type 2 diabetes mellitus.

The multivariate logistic regression models identified six independent significant risk factors of CKD (Table 3): (i) older age of 56–65 years (adjusted odds ratio [OR], 2.80; 95% CI, 1.59–4.93), 66–75 years (adjusted OR, 5.41; 95% CI, 2.97–9.88), and over 75 years (adjusted OR, 27.44; 95% CI, 13.51–55.73); (ii) retinopathy (adjusted OR, 3.41; 95% CI, 2.18–5.34); (iii) albuminuria (adjusted OR, 2.08; 95% CI, 1.43–3.02); (iv) haemoglobin A1c ≥ 7% (adjusted OR, 3.32; 95% CI, 2.20–5.01); (v) haemoglobin <12 g/dL in females or <13 g/dL in males (adjusted OR, 3.32; 95% CI, 2.20–5.01); (vi) uric acid>7.5 mg/dL (adjusted OR, 9.00; 95% CI, 5.82–13.92). The concordance (*c*) statistic or the area under the receiver operating characteristic (AuROC) curve of the final model was 0.87 (95% CI, 0.85–0.90), considered to have an excellent discrimination, in which the risk factors model explained 87.3% of the probability of CKD among T2DM patients (Fig. 2).

**Table 3:** Multivariable risk factors of CKD in patients with T2DM (n = 1,061).

Factors	Adjusted OR (95% CI)	*P* Value
Age, year		
<55	1.00 (Reference)	
56–65	2.80 (1.59–4.93)	<0.001
66–75	5.41 (2.97–9.88)	<0.001
>75	27.44 (13.51–55.73)	<0.001
Retinopathy		
No	1.00 (Reference)	
Yes	3.41 (2.18–5.34)	<0.001
Albuminuria		
No	1.00 (Reference)	
Yes	2.08 (1.43–3.02)	<0.001
Haemoglobin A1c, %		
<7	1.00 (Reference)	
≥7	3.32 (2.20–5.01)	<0.001
Haemoglobin, g/dL		
≥12 in females or ≥13 in males	1.00 (Reference)	
<12 in females or <13 in males	2.96 (2.07–4.23)	<0.001
Uric acid, mg/dL		
≤7.5	1.00 (Reference)	
>7.5	9.00 (5.82–13.92)	<0.001
C statistic (95% CI)	0.87 (0.85–0.90)	

Abbreviations: CI, confidence interval; CKD, chronic kidney disease; OR, Odds ratio; T2DM, type 2 diabetes mellitus.

### Sensitivity analyses

According to the different equations for estimating GFR < 60 mL/min/1.73 m^2^ (CKD-EPI equation for Asian population, the modification of diet in renal disease [MDRD] equation, and the Thai GFR equation; Supplementary Table S2), the Cohen’s kappa coefficient (κ) was 0.87–0.93, indicating close to perfect agreement between the prevalence of CKD using the CKD-EPI equation and the other proposed equations (Supplementary Table S3). Using the proposed eGFR equations, the overall prevalence of CKD was 21.4–27.7%, with the severity of 10.0–13.4%, 6.7–8.2%, 2.0–4.4%, and 0.6–1.6% for stages 3 A, 3B, 4, and 5, respectively (Supplementary Table S4, Fig. S2).

For risk factors associated with CKD, using the multiple imputation analysis, restricting the analysis by excluding patients with hyperfiltration (eGFR ≥120 mL/min/1.73 m^2^), and re-analysed risk factors of CKD using the proposed different eGFR equations did not alter the risk factors model (c-statistic, 0.87–0.88; Supplementary Tables S5, S6).

## Discussion

This study examined the burden of CKD in adult T2DM patients in a suburban community in Thailand. We found that CKD is a common diabetes-related complication among T2DM patients. Within a primary care setting, the estimated prevalence of CKD stages 3–5 (eGFR <60 mL/min/1.73 m^2^) in T2DM patients was 24.4% (95% CI, 21.9–27.0), with substantial variation by age and glycaemic control status. From a clinical perspective, risk factors for the development of CKD in our study can help inform the clinical decision-making process and the formation of the appropriate care strategy for T2DM patients. As such, our study can lay the foundation for routine surveillance for T2DM patients who are at high risk of CKD in the primary care setting.

The treatment of diabetes generally differs by CKD status because individuals without CKD are treated with oral antidiabetic drugs, while those with CKD receive insulin therapy. According to strategies targeting kidney-specific disease, T2DM patients in our study were more commonly prescribed renin-angiotensin system (RAS) inhibitors (59.0%), whereas the utilisation of these agents varied across diabetes care practices worldwide as 29.6–56.0%^22–25^. Despite an improvement in diabetes care over time, suboptimal glycaemic control remains observed in our study, with only 36.1% meeting the glycaemic goal of haemoglobin A1c < 7%, particularly those with CKD. We also found that T2DM patients with CKD were more likely to have diabetes-related complications including ischaemic heart disease, cerebrovascular disease, diabetic retinopathy, and albuminuria than those without CKD. Taken together, these figures are in line with previous nationwide reports in Thailand^26^.

Recently, large randomised controlled trials suggest that the use of sodium-glucose cotransporter 2 (SGLT-2) inhibitors or glucagon-like peptide 1 (GLP-1) receptor agonists shown to reduce the risk of CKD progression and improve kidney outcomes^27–30^. However, during the study period, the novelty of the new drug class of SGLT-2 inhibitors and GLP-1 receptor agonists were not available in the National Medicines Formulary in Thailand under the health benefits package. As such, further studies are needed on treatments modifying the risk of development of CKD among T2DM in the real-world primary care settings.

To our knowledge, our finding suggests a lower prevalence and is comparable to a national study of CKD in adult T2DM patients in Thailand found at 24.4% vs. 35.4%, respectively^17^. A similar trend in the prevalence of CKD was observed in elderly patients (>65 years) with T2DM—at 40.5% and 56.1% in our study and national level in Thailand, respectively^31^. Unlike urbanised areas, CKD rates among the T2DM patients in our study were comparable to those reported in previous studies of less urbanised communities or regional areas in Thailand^19,20^. According to the Global Burden Disease-CKD study, CKD due to diabetes accounted for 30.7% of CKD populations, in which T2DM was the only cause of CKD to illustration a substantial increase in the age-standardised rate (changed by 9.5% from 1990 to 2017)^32^. Globally, the overall prevalence of CKD among T2DM patients varied at 6.0–39.3% (our result found at 24.4%)^17,25,33–47^. These discrepancies across different settings may be attributed to the variations in diagnostic methods used and ethnicities such as the black race, which is associated with a greater rate of GFR decline^48^. Overall, our result parallels the global rates of diabetes populations, which are expected to occur lower than in the rural areas or less urbanised community^1^, suggesting that our findings have general relevance.

In this study, several diabetes-specific and general risk factors in the literature for CKD among T2DM patients were investigated (Supplementary Table S1). However, we did not find an association between hypertension or blood pressure and the risk of CKD among T2DM patients, which was reported in previous studies^18,21,39–42^. The lack of this relationship could be attributable to in part to increasing usage of RAS inhibitors for protection against kidney disease and improved blood pressure control in our diabetes practice. Moreover, since most of our study patients were already receiving antihypertensive agents, the lack of association between blood pressure and the risk of CKD is not surprising. Consequently, six independent significant risk factors of CKD were identified including older age (>55 years), retinopathy, albuminuria, haemoglobin A1c ≥ 7%, anaemia (haemoglobin <12 g/dL in females or <13 g/dL in males), and uric acid>7.5 mg/dL.

With respect to non-modifiable risk factors, managing elderly patients with T2DM is challenging, as this population has a high rate of comorbid conditions as also associated with a greater risk of developing CKD. Our findings showed that T2DM patients aged 56–65, 66–75, and>75 years had more than 2.8-fold, 5.4-fold, and 27.4-fold higher adjusted ORs for CKD, respectively. This result reaffirms that of previous studies that older age was associated with a higher risk of CKD among T2DM patients^33,37,39–42,44^. Cardiovascular disease, obesity, and multimorbidity via endothelial cell dysfunction and sympathetic nervous system activation resulting in increased atherosclerosis, hypertension, and progressive nephrosclerosis are believed to explain the mechanisms underlying older age and the risk of CKD^49,50^.

With respect to modifiable risk factors, glycaemic control was the most determinant of the development of diabetes-related complications and the risk of CKD in T2DM. Based on our findings, the presence of albuminuria, diabetic retinopathy, and poor glycaemic control (haemoglobin A1c ≥ 7%) are independent risk factors for the development of CKD among T2DM patients. Indeed, albuminuria and diabetic retinopathy are components of diabetes-related microvascular complications, especially in those with poor glycaemic control. These factors have been previously recognised as risk factors for the development of CKD in T2DM patients^18,37,39,41,42^. In concordance with previous reports^42,51^, our study demonstrates that anaemia, defined as haemoglobin <12 g/dL in females or <13 g/dL in males, commonly occurs in T2DM patients (38.5%), particularly in the elderly and those with more comorbid conditions. As expected, a significant association was observed that T2DM patients with anaemia had more than a 3.0-fold higher risk of CKD. Our finding corresponds well with previous studies that hyperuricemia is a strong independent risk factor of the development of CKD^52–55^. Evidence illustrates that the GFR deterioration is associated with progressive impairment in uric acid excretion, resulting in insulin resistance and hypertension. Experimental studies also revealed that increased serum uric acid concentrations are associated with kidney damage via stimulating RAS activity and promoting endothelial damage along with oxidative stress^56–58^.

This study was based on patient-level information by the retrieval and linking of routinely collecting data, which provide detailed primary care practice on diabetes and kidney care. Our study delivers previously unrecognised data on the prevalence and risk factors of CKD among T2DM in a suburban community through a comprehensive process and rigorous statistical approaches. Moreover, the consistency of findings was observed based on our set of sensitivity analyses.

However, our findings should be interpreted in the context of certain limitations. First, the causal inference and the chronicity of the observations must be considered because our findings were based on the observational cross-sectional nature of the analyses. Moreover, longitudinal data were not obtained in this study; thus, temporal trends in prevalence and dynamic risk prediction for CKD among T2DM patients cannot be established over time. Second, this study was conducted within a single centre and was limited by the unique organisation of the Sansai Hospital, the suburban community care protocol implemented throughout the primary care unit and village health volunteers of this community. Accordingly, the generalisability of our finding to other T2DM populations and healthcare settings other than in primary care practice in Thailand is uncertain and warrants further study. Third, although we performed a series of sensitivity analyses using different equations for estimating GFR, misclassification (potential errors relating to CKD staging) is possible because eGFR alone is insufficient to evaluate kidney function, particularly in cases of advanced CKD. Moreover, urinary protein tests were not routinely available in our primary care practice. Therefore, detection bias should be noticed as it was not considered in our definition of CKD. Finally, contextual factors related to diabetes control including, patient comorbidities, health behaviours (e.g. dietary intake and physical activity), mental health problems (e.g. depression, social support, and coping skills), and social determinants of health (education and literacy, income and social status, physical environments, employment status, and health inequity) were obtained. Moreover, novel biomarkers and relevant inflammatory markers were not available in our primary diabetic care practices. In this circumstance, the residual risk factors may also influence the prevalence and risk factors of CKD among T2DM patients. However, the risk factors for development CKD in our study illustrated an excellent performance of the model prediction in terms of discriminative ability, which explained 87.3% of the probability of CKD among T2DM patients.

Due to rapid urbanisation and the dramatic increase in the elderly population, our findings support the well-recognised fact that routine surveillance is mandatory to prevent the development of ESRD to decrease the healthcare burden and costs-related to RRT treatment. This study may also contribute to improved diabetes care management by the early identification and targeting of T2DM patients who are at high risk of developing CKD. Further studies are needed to assess the utility of integrating the clinical predictive factors of CKD among T2DM patients as a part of routine diabetes care and call for strategic goals and actions upon their recognition to reduce the CKD incidence or slow CKD progression. Ultimately, long-term holistic healthcare services in a primary care practice should be targeted based on multimorbidity concepts, particularly in the elderly, to reduce the prevalence of CKD and mitigate the large public health effect of CKD in T2DM patients.

In summary, here we found a relatively high prevalence of CKD among T2DM patients in a suburban community in Thailand, particularly in elderly patients and those with diabetes complications related to poor glycaemic control. Our study also underscores an important opportunity to identify T2DM patients who are at high risk of CKD through readily available and routinely obtained factors in the primary care setting. Early identification may help optimise care and prevention programs for these populations.

## Methods

### Study design and patient population

This retrospective cross-sectional study used a cohort of T2DM patients from the Sansai Hospital suburban community in northern Thailand from October 1, 2016 through September 30, 2017. Data were obtained from the electronic health records from the Sansai Hospital database along with the routine medical records from the Sansai primary care settings. The datasets were linked and merged comprising: (i) outpatient and inpatient data; (ii) administrative data on pharmacy dispensing and laboratory support system; (iii) primary care practice on diabetes and kidney care, with patient-level detail on socio-demographic factors, clinical characteristics, and routine diabetes clinical examination findings. An external consensus panel of two health information professionals reviewed, verified, and validated the datasets for high-quality data collection system and to limit the quantity of missing data.

The study was approved by the institutional review boards of Chiang Mai Provincial Public Health Office and the Hospital Authority of the Sansai Hospital. Informed consent in this study was waived owing to the retrospective nature of our study and de-identification of the patient information which has been accepted and allowed by the institutional review boards of Chiang Mai Provincial Public Health Office. The study protocol was conducted according to the Declaration of Helsinki and reported in line with the Strengthening the Reporting of Observational Studies in Epidemiology (STROBE) Statement guidelines for cross-sectional studies (Appendix in the Supplement)^59^.

Patients eligible for inclusion in this study included those: (i) who were aged 18 years or older; (ii) who were diagnosed with T2DM; (iii) for whom eGFR values had been documented more than twice from their index date. The exclusion criteria were: (i) having received chronic dialysis treatment or kidney transplantation; (ii) incomplete data on glycaemic control; and (iii) currently pregnancy or breastfeeding.

### Outcome: Kidney function measures

According to the Kidney Disease: Improving Global Outcomes (KDIGO) CKD Work Group, serum creatinine measurements were used to calculate eGFR based on the CKD-EPI equation^60,61^. Theoretically, eGFR alone is insufficient to indicate the presence of CKD, particularly in less advanced stages. Thus, we considered only those with advanced CKD stages (stages 3–5): eGFR values of <60 mL/min/1.73 m^2^ on more than days apart from their index date. Patients were classified as having CKD stage 3 A, 3B, 4, or 5 if they had an eGFR value of 45–59, 30–44, 15–29, or <15 mL/min/1.73 m^2^, respectively, since urinary protein tests were not available in the primary care practice. However, to be comprehensive and reflect routine clinical practice, patients for whom urine dipstick measurements or urine albumin-creatinine ratios (UACR) were available were evaluated for albuminuria (≥1+ in dipstick studies or>30 mg/g in UACR studies).

### Candidate risk factors

Potential risk factors of CKD among T2DM patients were recognised based on a comprehensive review and the list of risk factors that are routinely and readily available at primary care practice^62,63^. Candidate risk factors included: (i) socio-demographic characteristics (age, sex, BMI, smoking status, alcohol consumption, insurance status, medical history [hypertension, coronary artery disease, cerebrovascular disease, retinopathy, and albuminuria], blood pressure, and duration of diabetes); (ii) laboratory values (serum creatinine, fasting plasma glucose, haemoglobin A1c, haemoglobin, uric acid, and lipid profiles); and (iii) medication treatment (glycaemic control, antihypertensive agents, lipid-lowering agents, antiplatelet agents, and anti-gout agents).

### Sample size

The sample size was estimated based on two parameters: (i) national and international prevalence of CKD (eGFR <60 mL/min/1.73 m^2^) among T2DM patients with a range of 6.0–39.3%;^17,18,25,33–47^ and (ii) the list of risk factors associated with CKD among T2DM patients from the previous studies including older age^33,37,39–42,44^, sex^44^, BMI^37^, smoking^37^, albuminuria^37^, retinopathy^39,41,42^, hypertension^39,41^, cerebrovascular disease^41^, anaemia^42,51^, duration of diabetes^18,33,39,40,44^, blood pressure^18,33,40,42^, haemoglobin A1c^18,39^, serum uric acid^52,53,55^, and serum lipid profiles^33,37,39^. To compensate for 10% missing data, at least 1,001 T2DM patients were included for the present study to ensure a power of 80% and a 0.05 type I error.

### Statistical analysis

Descriptive data are summarised as the number with percentage for categorical variables and mean ± SD or medians with IQR as appropriate. Difference between CKD status (eGFR<60 vs. ≥60 mL/min/1.73 m^2^) were assessed using Fisher’s exact test and unpaired t-test or Wilcoxon rank-sum test for categorical and continuous data, respectively.

Prevalence rates estimated with 95% CIs of CKD (eGFR <60 mL/min/1.73 m^2^), as well as CKD stage (stage 3–5), were analysed according to sociodemographic (age and sex) and glycaemic control (haemoglobin A1c). To identify the candidate risk factors, the crude association between patient characteristics and CKD was assessed through the univariable logistic regression models. Subsequently, risk factors with a *P*-value less than 0.100 were then included in the multivariate logistic regression analysis with the stepwise backward method. The final model was also determined for multicollinearity by investigation of the variance inflation factors of the risk factors within the multivariable model.

The effect estimates of final risk factors model for CKD among T2DM patients were expressed as ORs with corresponding 95% CI. Moreover, the *c*-statistic or the AuROC curve was performed to indicate the ability of a final model to distinguish patients with or without CKD (eGFR <60 vs. ≥60 mL/min/1.73 m^2^). A *c*-statistic more than 0.7 indicate acceptable discriminative of the model^64^. Variables with more than 20% of the values were excluded from the primary analysis; however, a multiple imputation method was performed in the sensitivity analysis. All analyses were performed using Stata version 14.0 (StataCorp LP, TX). Two-tailed tests with values of *P* < 0.05 were considered statistically significant.

### Sensitivity analyses

Additional analyses were further assessed to address the robustness of our findings. For the prevalence of CKD, the different equations for estimating GFR < 60 mL/min/1.73 m^2^ were performed using the CKD-EPI equation for Asian population^65^, MDRD equation^66^, and Thai GFR equation^67^ The agreement of prevalence of CKD using the CKD-EPI equation and other proposed study equations for estimating GFR was estimated using the κ statistic (>0.8 indicates almost perfect agreement)^68^.

For risk factors associated with CKD, sensitivity analyses were conducted by (i) using the multiple imputation analysis to account for missing values; (ii) restricting the analysis by excluding patients with hyperfiltration (eGFR ≥120 mL/min/1.73 m^2^) that may contribute to the progression of kidney disease among diabetes patients^69^; and (iii) re-analysing the risk factors of CKD ( < 60 mL/min/1.73 m^2^) using the three different eGFR equations as described above.

## Supplementary information

Supplementary Information.

## Data Availability

Data are available from the authors upon reasonable request and with permission of the Hospital Authority of the Sansai Hospital, Chiang Mai Province, Thailand.
