# Supplementary material for: Prevalence and Risk Factors of Chronic Kidney Disease among Type 2 Diabetes Patients: A Cross-Sectional Study in Primary Care Practice
Source: Sci Rep. 2020 Apr 10;10:6205. doi: 10.1038/s41598-020-63443-4 (PMC7148316; doi:10.1038/s41598-020-63443-4)
Supplement: Supplementary file 1 — Supplementary Information. [file 41598_2020_63443_MOESM1_ESM.pdf]

Supplementary Information

Prevalence and Risk Factors of Chronic Kidney Disease among Type 2 Diabetes Patients: A Cross-Sectional Study in Primary Care Practice

Janjira Jitraknatee, MD[1], Chidchanok Ruengorn, PhD (ORCID iD: orcid.org/0000-0001-7927-1425)[2,3]; Surapon Nochaiwong, PharmD (ORCID iD: orcid.org/0000-0003-1100-7171)[2,3*]

Affiliations:

[1]Kidney Center, Sansai Hospital, Chiang Mai 50290, Thailand

[2]Department of Pharmaceutical Care, Faculty of Pharmacy, Chiang Mai University, Chiang Mai 50200, Thailand

[3]Pharmacoepidemiology and Statistics Research Center (PESRC), Faculty of Pharmacy, Chiang Mai University, Chiang Mai 50200, Thailand

***Correspondence and requests for materials:**

Surapon Nochaiwong, PharmD, Department of Pharmaceutical Care, Faculty of Pharmacy, Chiang Mai University, Chiang Mai 50200, Thailand, Phone: 66899973365, Fax: 6653222741, Email: surapon.nochaiwong@gmail.com

Supplementary Online Content

**Table S1**	Univariable Risk Factors of CKD in Patients with T2DM	S3
**Table S2**	Study Equations for Estimating GFR Expressed	S7
**Table S3**	Agreement Between Study Equations Used to Diagnosed CKD (eGFR<60 mL/min/1.73 m^2)	S8
**Table S4**	Age-, Sex- and Glycaemic Control-Specific Prevalence of CKD in Patients with T2DM According to Study Equations for Estimating GFR Expressed	S9
**Table S5**	Sensitivity Analyses of Risk Factors of CKD in Patients with T2DM According to Equation for Estimating GFR Expressed	S12
**Table S6**	Sensitivity Analyses of Risk Factors of CKD in Patients with T2DM According to Type of analysis	S13
**Figure S1**	Study Flow on the Selection of Eligible Patients	S14
**Figure S2**	Age, Sex and Glycaemic Control Adjusted Prevalence Rates of CKD in Patients with T2DM According to Equation for Estimating GFR Expressed	S15
**eReferences**		S17
**Appendix**	STROBE Statement	S18

Table S1. Univariable Risk Factors of CKD in Patients with T2DM

Factors	Crude OR (95% CI)	*P* value
Sex		
Male	1.00 (Reference)	
Female	0.99 (0.75 – 1.31)	9.939
Age, year		
<55	1.00 (Reference)	
56-65	3.01 (1.83 – 4.96)	<0.001
66-75	5.39 (3.22 – 9.04)	<0.001
>75	18.12 (10.01 – 32.78)	<0.001
BMI, kg/m^2		
<18.5	0.64 (0.36 – 1.15)	0.138
18.5-22.9	1.00 (Reference)	
23-24.9	0.89 (0.60 – 1.32)	0.578
≥25	0.63 (0.46 – 0.87)	0.006
Smoking status		
Never	1.00 (Reference)	
Former	1.13 (0.54 – 2.36)	0.749
Current	0.76 (0.44 – 1.30)	0.320
Alcohol consumption		
Never	1.00 (Reference)	
Former	0.76 (0.36 – 1.62)	0.484
Current	0.65 (0.42 – 0.99)	0.044
Insurance status		
UCS by NHSO	1.00 (Reference)	
CSMBS	0.97 (0.64 – 1.47)	0.886
SSS/others	0.78 (0.50 – 1.21)	0.265
Hypertension		
No	1.00 (Reference)	
Yes	2.31 (1.51 – 3.53)	<0.001
CAD		
No	1.00 (Reference)	
Yes	3.01 (1.93 – 4.69)	<0.001
CBVD		
No	1.00 (Reference)	
Yes	2.54 (1.59 – 4.05)	<0.001
Retinopathy		
No	1.00 (Reference)	
Yes	3.75 (2.63 – 5.35)	<0.001
Albuminuria		
No	1.00 (Reference)	
Yes	2.59 (1.93 – 3.48)	<0.001

Abbreviations: BMI, body mass index; CAD, coronary artery disease; CBVD, cerebrovascular disease; CI, confidence interval; CKD, chronic kidney disease; CSMBS, Civil Servant Medical Benefit Scheme; NHSO, National Health Security Office; OR, odds ratio; SSS, Social Security Scheme; T2DM, type 2 diabetes mellitus; UCS, Universal Coverage Scheme.

Table S1. Univariable Risk Factors of CKD in Patients with T2DM (Continued)

**Factors**	**Crude OR (95% CI)**	***P* value**
Systolic BP, mmHg		
<140	1.00 (Reference)	
≥140	1.35 (1.02 – 1.80)	0.038
Diastolic BP, mmHg		
<90	1.00 (Reference)	
≥90	0.82 (0.57 – 1.18)	0.290
Long-standing diabetes (>10 years)		
No	1.00 (Reference)	
Yes	2.02 (1.52 – 2.69)	<0.001
Fasting plasma glucose, mg/dL		
<100	1.00 (Reference)	
100-125	1.15 (0.69 – 1.92)	0.587
≥126	0.86 (0.63 – 1.19)	0.376
Haemoglobin A_{1c}, %		
<7	1.00 (Reference)	
≥7	2.62 (1.89 – 3.62)	<0.001
Haemoglobin, g/dL		
≥12 in females or ≥13 in males	1.00 (Reference)	
<12 in females or <13 in males	3.93 (2.94 – 5.26)	<0.001
Uric acid, mg/dL		
≤7.5	1.00 (Reference)	
>7.5	7.80 (5.46 – 11.13)	<0.001
Total cholesterol, mg/dL		
≤200	1.00 (Reference)	
>200	0.95 (0.71 – 1.26)	0.705
Triglycerides, mg/dL		
≤150	1.00 (Reference)	
>150	1.11 (0.84 – 1.47)	0.454
LDL-C, mg/dL		
≤100	1.00 (Reference)	
>100	1.18 (0.89 – 1.58)	0.254
HDL-C, mg/dL		
≥50 in females or ≥40 in males	1.00 (Reference)	
<50 in females or <40 in males	1.25 (0.94 – 1.65)	0.123
Non-HDL-C, mg/dL		
≤130	1.00 (Reference)	
>130	1.13 (0.84 – 1.51)	0.416

Abbreviations: BP, blood pressure; CI, confidence interval; CKD, chronic kidney disease; HDL-C, high density lipoprotein cholesterol; LDL-C, low density lipoprotein cholesterol; OR, odds ratio; T2DM, type 2 diabetes mellitus.

Table S1. Univariable Risk Factors of CKD in Patients with T2DM (Continued)

Factors	Crude OR (95% CI)	*P* value
Glycaemic control		
Diet only	1.00 (Reference)	
Metformin only	0.36 (0.21 – 0.61)	<0.001
Sulfonylurea only†	1.73 (1.03 – 2.91)	0.038
Metformin plus sulfonylurea	0.32 (0.20 – 0.53)	<0.001
Other oral antidiabetic drugs‡	3.89 (0.34 – 44.34)	0.274
Insulin only	1.99 (1.11 – 3.56)	0.020
Insulin plus metformin	0.26 (0.10 – 0.66)	0.005
Insulin plus sulfonylurea	1.58 (0.69 – 3.63)	0.281
Insulin plus metformin plus sulfonylurea	0.38 (0.17 – 0.84)	0.016
ACEIs/ARBs		
No	1.00 (Reference)	
Yes	0.88 (0.66 – 1.16)	0.359
Beta-blockers		
No	1.00 (Reference)	
Yes	2.61 (1.83 – 3.74)	<0.001
CCBs		
No	1.00 (Reference)	
Yes	1.16 (0.88 – 1.54)	0.298
Loop diuretic		
No	1.00 (Reference)	
Yes	6.06 (3.78 – 9.71)	<0.001
Thiazide		
No	1.00 (Reference)	
Yes	1.00 (0.57 – 1.72)	0.987
Other antihypertensive agents§		
No	1.00 (Reference)	
Yes	3.21 (1.86 – 5.55)	<0.001
No. of antihypertensive therapy		
<3	1.00 (Reference)	
≥3	2.81 (1.89 – 4.19)	<0.001
Statins		
No	1.00 (Reference)	
Yes	1.15 (0.86 – 1.52)	0.339
Fibrates		
No	1.00 (Reference)	
Yes	0.98 (0.58 – 1.66)	0.946

†Includes glibenclamide, glipizide.

‡Includes pioglitazone only, metformin plus pioglitazone, metformin plus glipizide plus pioglitazone.

§Includes hydralazine, methydopa, doxazosin.

Abbreviations: ACEIs/ARBs, angiotensin-converting enzyme inhibitors/angiotensin II receptor blockers; CCBs, calcium channel blockers; CI, confidence interval; CKD, chronic kidney disease; T2DM, type 2 diabetes mellitus.

Table S1. Univariable Risk Factors of CKD in Patients with T2DM (Continued)

Factors	Crude OR (95% CI)	*P* value
Antiplatelet agents		
No	1.00 (Reference)	
Yes	1.45 (1.10 – 1.92)	0.008
Allopurinol		
No	1.00 (Reference)	
Yes	2.45 (0.90 – 6.64)	0.079
Colchicine		
No	1.00 (Reference)	
Yes	2.95 (1.33 – 6.54)	0.008

Abbreviations: CI, confidence interval; CKD, chronic kidney disease; T2DM, type 2 diabetes mellitus.

Table S2. Study Equations for Estimating GFR Expressed

Sex	Scr (mg/dL)	Equation†
**CKD-EPI Study Equation[1]**		
Female	≤0.7	GFR = 144 x $(Scr/0.7)^{-0.329}$ $x(0.993)^{Age}$
Female	>0.7	GFR = 144 x $(Scr/0.7)^{-1.209}$ $x(0.993)^{Age}$
Male	≤0.9	GFR = 144 x $(Scr/0.9)^{-0.411}$ $x(0.993)^{Age}$
Male	>0.9	GFR = 144 x $(Scr/0.9)^{-1.209}$ $x(0.993)^{Age}$
**CKD-EPI Study Equation for Asian[2]**		
Female	≤0.7	GFR = 151 x $(Scr/0.7)^{-0.328}$ $x(0.993)^{Age}$
Female	>0.7	GFR = 151 x $(Scr/0.7)^{-1.210}$ $x(0.993)^{Age}$
Male	≤0.9	GFR = 149 x $(Scr/0.9)^{-0.412}$ $x(0.993)^{Age}$
Male	>0.9	GFR = 149 x $(Scr/0.9)^{-1.210}$ $x(0.993)^{Age}$
**MDRD Study Equation[3]**		
Female	All	GFR = 175 x $Scr^{-1.154}$ x $Age^{-0.203}$ x 0.742
Male	All	GFR = 175 x $Scr^{-1.154}$ x $Age^{-0.203}$
**Thai GFR Equation[4]**		
Female	All	GFR = 375.5 x $Scr^{-0.848}$ x $Age^{-0.364}$ x 0.712
Male	All	GFR = 375.5 x $Scr^{-0.848}$ x $Age^{-0.364}$

†Conversion factors for units: GFR in mL/min/1.73 m2 to mL/s/1.73 m2, ×0.0167; SCr in mg/dL to µmol/L, ×88.4.

Abbreviations: CKD-EPI, the Chronic Kidney Disease Epidemiology Collaboration; GFR, glomerular filtration rate; MDRD, the modification of diet in renal disease.

Table S3. Agreement Between Study Equations Used to Diagnosed CKD (eGFR<60 mL/min/1.73 m^2)

**eGFR Equation**	**Kappa coefficient of Agreement with the CKD-EPI Equation (95% CI)**
CKD-EPI Equation for Asian	0.93 (0.91-0.96
MDRD Equation	0.90 (0.87-0.93)
Thai GFR Equation	0.87 (0.84-0.90)

Abbreviations: CI, confidence interval; CKD, chronic kidney disease; CKD-EPI, the Chronic Kidney Disease Epidemiology Collaboration; eGFR, estimated glomerular filtration; MDRD, the modification of diet in renal disease.

Table S4. Age-, Sex- and Glycaemic Control-Specific Prevalence of CKD in Patients with T2DM According to Study Equations for Estimating GFR Expressed: CKD-EPI Equation for Asian

**Characteristics**	**N**	**Stage 3A**		**Stage 3B**		**Stage 4**		**Stage 5**		**Overall CKD**	
		**Cases**	**Prevalence Estimated (95% CI)**	**Cases**	**Prevalence Estimated (95% CI)**	**Cases**	**Prevalence Estimated (95% CI)**	**Cases**	**Prevalence Estimated (95% CI)**	**Cases**	**Prevalence Estimated (95% CI)**
**Age, year**											
<45	68	1	1.5 (0.2-9.8)	1	1.4 (0.2-9.8)	1	1.5 (0.2-9.8)	0	0.0	3	4.4 (1.4-12.9)
45-55	219	15	6.8 (4.2-11.1)	3	1.4 (0.4-4.2)	1	0.4 (0.1-3.2)	3	1.4 (0.4-4.2)	22	10.0 (6.7-14.8)
56-65	439	30	6.8 (4.8-9.6)	25	5.7 (3.9-8.3)	16	3.6 (2.2-5.9)	8	1.8 (0.9-3.6)	79	18.0 (14.7-21.9)
66-75	262	35	13.4 (9.7-18.0)	23	8.8 (5.9-12.9)	15	5.7 (3.5-9.3)	5	1.9 (0.8-4.5)	78	29.8 (24.5-35.6)
>75	106	28	26.4 (18.9-35.6)	21	19.8 (13.2-28.5)	9	8.5 (4.4-15.6)	1	0.9 (0.1-6.4)	59	55.7 (46.0-64.9)
**Sex**											
Male	469		9.8 (7.4-12.8)	33	7.0 (5.0-9.7)	14	3.0 (1.8-5.0)	6	1.3 (0.6-2.8)	99	21.1 (17.6-25.0)
Female	625		10.1 (7.9-12.7)	40	6.4 (4.7-8.6)	28	4.5 (3.1-6.4)	11	1.8 (1.0-3.2)	142	22.7 (19.6-26.2)
**Glycaemic control, haemoglobin A_{1c} (%)**											
<6	103	7	6.8 (3.2-13.6)	7	6.8 (3.2-13.6)	4	3.9 (1.4-9.9)	2	1.9 (0.5-7.5)	20	19.4 (12.8-28.2)
6-6.9	292	21	7.2 (4.7-10.8)	6	2.0 (0.9-4.5)	2	0.7 (0.2-2.7)	3	1.0 (0.3-3.1)	32	11.0 (7.8-15.1)
7-7.9	241	16	6.6 (4.1-10.6)	8	3.3 (1.7-6.5)	6	2.5 (1.1-5.4)	1	0.4 (0.1-2.9)	31	12.9 (9.2-17.7)
≥8	458	65	14.2 (11.3-17.7)	52	11.4 (8.8-14.6)	30	6.6 (4.6-9.2)	11	2.4 (1.3-4.3)	158	34.5 (30.3-39.0)
**All**	**1,094**	**109**	**10.0 (8.3-11.9)**	**71**	**6.7 (5.3-8.3)**	**42**	**3.8 (0.3-5.2)**	**17**	**1.6 (1.0-2.5)**	**241**	**22.0 (19.7-24.6)**

Abbreviations: CI, confidence interval; CKD. chronic kidney disease; CKD-EPI, the Chronic Kidney Disease Epidemiology Collaboration; GFR, glomerular filtration rate; T2DM, type 2 diabetes mellitus.

Table S4. Age-, Sex- and Glycaemic Control-Specific Prevalence of CKD in Patients with T2DM According to Study Equations for Estimating GFR Expressed: MDRD Equation (Continued)

Characteristics	N	Stage 3A		Stage 3B		Stage 4		Stage 5		Overall CKD	
		Cases	Prevalence Estimated (95% CI)	Cases	Prevalence Estimated (95% CI)	Cases	Prevalence Estimated (95% CI)	Cases	Prevalence Estimated (95% CI)	Cases	Prevalence Estimated (95% CI)
**Age, year**											
<45	68	0	0.0	2	2.9 (0.7-11.1)	1	1.5 (0.2-9.8)	0	0.0	3	4.4 (1.4-12.9)
45-55	219	16	7.3 (4.5-11.6)	11	5.8 (2.8-8.8)	1	0.4 (0.1-3.2)	3	1.4 (0.4-4.2)	31	14.2 (10.1-19.4)
56-65	439	54	12.3 (9.5-15.7)	31	7.1 (5.0-9.9)	17	3.9 (2.4-6.1)	10	2.2 (1.2-4.2)	112	25.5 (21.6-29.8)
66-75	262	47	17.9 (13.7-23.1)	25	9.5 (6.5-13.8)	18	6.9 (4.4-10.6)	5	1.9 (0.8-4.5)	95	36.2 (30.6-42.3)
>75	106	30	28.3 (20.5-37.6)	21	19.8 (13.2-28.5)	11	10.4 (5.8-17.8)	0	0.0	62	58.5 (48.8-67.5)
**Sex**											
Male	469	65	13.8 (11.0-17.3)	38	8.1 (5.9-10.9)	18	3.8 (2.4-6.0)	6	1.3 (0.6-2.8)	127	27.1 (23.2-31.3)
Female	625	82	13.1 (10.7-16.0)	52	8.3 (6.4-10.8)	30	4.8 (3.4-6.8)	12	1.9 (1.1-3.4)	176	28.2 (24.8-31.8)
**Glycaemic control, haemoglobin A_{1c} (%)**											
<6	103	10	9.7 (5.3-17.2)	6	5.8 (2.6-12.4)	6	5.8 (2.6-12.4)	1	1.0 (0.1-6.6)	23	22.3 (15.3-31.4)
6-6.9	292	35	12.0 (8.7-16.2)	10	3.4 (1.8-6.2)	2	0.7 (0.2-2.7)	3	1.0 (0.3-3.1)	50	17.1 (13.2-21.9)
7-7.9	241	24	10.0 (6.8-14.4)	9	3.7 (2.0-7.0)	8	3.3 (1.7-6.5)	1	0.4 (0.1-2.9)	42	17.4 (13.1-22.8)
≥8	458	78	17.0 (13.8-20.8)	65	14.2 (11.3-17.7)	32	7.0 (5.0-9.7)	13	2.8 (1.6-4.8)	188	41.0 (36.6-45.6)
**All**	**1,094**	**147**	**13.4 (11.5-15.6)**	**90**	**8.2 (6.7-10.0)**	**48**	**4.4 (3.3-5.8)**	**18**	**1.6 (1.0-2.6)**	**303**	**27.7 (25.1-30.4)**

Abbreviations: CI, confidence interval; CKD. chronic kidney disease; GFR, glomerular filtration rate; MDRD, the modification of diet in renal disease; T2DM, type 2 diabetes mellitus.

Table S4. Age-, Sex- and Glycaemic Control-Specific Prevalence of CKD in Patients with T2DM According to Study Equations for Estimating GFR Expressed: Thai GFR Equation (Continued)

Characteristics	N	Stage 3A		Stage 3B		Stage 4		Stage 5		Overall CKD	
		Cases	Prevalence Estimated (95% CI)	Cases	Prevalence Estimated (95% CI)	Cases	Prevalence Estimated (95% CI)	Cases	Prevalence Estimated (95% CI)	Cases	Prevalence Estimated (95% CI)
**Age, year**											
<45	68	1	1.5 (0.2-9.8)	1	1.5 (0.2-9.8)	0	0.0	0	0.0	2	2.9 (0.7-11.1)
45-55	219	14	6.4 (3.8-10.5)	1	0.4 (0.1-3.2)	3	1.4 (0.4-4.2)	1	0.4 (0.1-3.2)	19	8.7 (5.6-13.2)
56-65	439	45	10.2 (7.7-13.5)	24	5.5 (3.7-8.0)	9	2.0 (1.1-3.9)	4	0.9 (0.3-2.4)	82	18.7 (15.3-22.6)
66-75	262	40	15.3 (11.4-20.2)	24	9.2 (6.2-13.3)	9	3.4 (1.8-6.5)	2	0.8 (0.2-3.0)	75	28.6 (23.5-34.4)
>75	106	30	28.3 (20.5-37.6)	25	23.6 (16.4-32.6)	1	0.9 (0.1-6.4)	0	0.0	56	52.8 (43.3-62.2)
**Sex**											
Male	469	44	9.4 (7.0-12.4)	23	4.9 (3.3-7.3)	4	0.8 (0.3-2.2)	2	0.4 (0.1-1.7)	73	15.6 (12.6-19.1)
Female	625	86	13.8 (11.3-16.7)	52	8.3 (6.4-10.8)	18	2.9 (1.8-4.5)	5	0.8 (0.3-1.9)	161	25.8 (22.5-29.3)
**Glycaemic control, haemoglobin A_{1c} (%)**											
<6	103	27	10.7 (6.0-18.3)	4	4.8 (2.0-11.2)	2	2.9 (0.9-8.7)	1	1.0 (0.1-6.6)	20	19.4 (12.8-28.2)
6-6.9	292	11	9.2 (6.4-13.2)	5	1.4 (0.5-3.6)	3	0.7 (0.2-2.7)	1	0.3 (0.0-2.4)	34	11.6 (8.4-15.9)
7-7.9	241	16	6.6 (4.1-10.6)	10	4.1 (2.2-7.6)	2	0.8 (0.2-3.3)	0	0.0	28	11.6 (8.1-16.3)
≥8	458	76	16.6 (13.4-20.3)	56	12.2 (9.5-15.6)	15	3.3 (2.0-5.4)	5	1.1 (0.4-2.6)	152	33.2 (29.0-37.6)
**All**	**1,094**	**130**	**11.9 (10.1-13.9)**	**75**	**6.8 (5.5-8.5)**	**22**	**2.0 (1.3-3.0)**	**7**	**0.6 (0.3-1.3)**	**234**	**21.4 (19.0-23.9)**

Abbreviations: CI, confidence interval; CKD. chronic kidney disease; GFR, glomerular filtration rate; T2DM, type 2 diabetes mellitus.

Table S5. Sensitivity Analyses of Risk Factors of CKD in Patients with T2DM According to Equation for Estimating GFR Expressed (n=1,061)

Factors	CKD-EPI Equation[1]		CKD-EPI Equation for Asian[2]		MDRD Equation[3]		Thai GFR Equation[4]	
	Adjusted OR (95% CI)	*P* Value	Adjusted OR (95% CI)	*P* Value	Adjusted OR (95% CI)	*P* Value	Adjusted OR (95% CI)	*P* Value
Age, year								
<55	1.00 (Reference)		1.00 (Reference)		1.00 (Reference)		1.00 (Reference)	
56-65	2.80 (1.59 – 4.93)	<0.001	2.23 (1.26 – 3.97)	0.006	2.62 (1.58 – 4.34)	<0.001	3.02 (1.65 – 5.52)	<0.001
66-75	5.41 (2.97 – 9.88)	<0.001	4.63 (2.53 – 8.48)	<0.001	4.54 (2.64 – 7.80)	<0.001	5.26 (2.80 – 9.88)	<0.001
>75	27.44 (13.51 – 55.73)	<0.001	19.33 (9.62 – 38.84)	<0.001	14.21 (7.46 – 27.01)	<0.001	18.84 (9.24 – 38.42)	<0.001
Retinopathy								
No	1.00 (Reference)		1.00 (Reference)		1.00 (Reference)		1.00 (Reference)	
Yes	3.41 (2.18 – 5.34)	<0.001	3.40 (2.17 – 5.32)	<0.001	3.14 (2.04 – 4.82)	<0.001	2.77 (1.78 – 4.31)	<0.001
Albuminuria								
No	1.00 (Reference)		1.00 (Reference)		1.00 (Reference)		1.00 (Reference)	
Yes	2.08 (1.43 – 3.02)	<0.001	2.33 (1.58 – 3.42)	<0.001	1.93 (1.38 – 2.72)	<0.001	2.27 (1.55 – 3.31)	<0.001
Haemoglobin A_{1c}, %								
<7	1.00 (Reference)		1.00 (Reference)		1.00 (Reference)		1.00 (Reference)	
≥7	3.32 (2.20 – 5.01)	<0.001	2.81 (1.86 – 4.26)	<0.001	2.49 (1.72 – 3.59)	<0.001	2.31 (1.55 – 3.45)	<0.001
Haemoglobin, g/dL								
≥12 (F) or ≥13 (M)	1.00 (Reference)		1.00 (Reference)		1.00 (Reference)		1.00 (Reference)	
<12 (F) or <13 (M)	2.96 (2.07 – 4.23)	<0.001	2.95 (2.05 – 4.23)	<0.001	2.67 (1.92 – 3.71)	<0.001	3.00 (2.10 – 4.27)	<0.001
Uric acid, mg/dL								
≤7.5	1.00 (Reference)		1.00 (Reference)		1.00 (Reference)		1.00 (Reference)	
>7.5	9.00 (5.82 – 13.92)	<0.001	7.55 (4.93 – 11.57)	<0.001	8.26 (5.43 – 12.56)	<0.001	5.56 (3.67 – 8.41)	<0.001
C statistic (95% CI)	0.87 (0.85 – 0.90)		0.87 (0.85 – 0.90)		0.87 (0.85 – 0.89)		0.87 (0.85 – 0.89)	

Abbreviations: CI, confidence interval; CKD, chronic kidney disease; CKD-EPI, the Chronic Kidney Disease Epidemiology Collaboration; F, female; GFR, glomerular filtration rate; M, male; MDRD, the modification of diet in renal disease; OR, Odds ratio; T2DM, type 2 diabetes mellitus.

Table S6. Sensitivity Analyses of Risk Factors of CKD in Patients with T2DM According to Type of analysis

Factors	Primary Analysis (n=1,061)		Multiple Imputation Analysis (n=1,094)		Excluding Patients with Hyperfiltration (n=1,038)†	
	Adjusted OR (95% CI)	*P* Value	Adjusted OR (95% CI)	*P* Value	Adjusted OR (95% CI)	*P* Value
Age, year						
<55	1.00 (Reference)		1.00 (Reference)		1.00 (Reference)	
56-65	2.80 (1.59 – 4.93)	<0.001	3.05 (1.73 – 5.37)	<0.001	2.59 (1.46 – 4.58)	0.001
66-75	5.41 (2.97 – 9.88)	<0.001	5.76 (3.15 – 10.51)	<0.001	4.99 (2.73 – 9.15)	<0.001
>75	27.44 (13.51 – 55.73)	<0.001	27.75 (13.78 – 55.91)	<0.001	25.24 (12.39 – 51.45)	<0.001
Retinopathy						
No	1.00 (Reference)		1.00 (Reference)		1.00 (Reference)	
Yes	3.41 (2.18 – 5.34)	<0.001	3.51 (2.26 – 5.48)	<0.001	3.35 (2.14 – 5.24)	<0.001
Albuminuria						
No	1.00 (Reference)		1.00 (Reference)		1.00 (Reference)	
Yes	2.08 (1.43 – 3.02)	<0.001	2.08 (1.44 – 3.01)	<0.001	2.09 (1.44 – 3.04)	<0.001
Haemoglobin A_{1c}, %						
<7	1.00 (Reference)		1.00 (Reference)		1.00 (Reference)	
≥7	3.32 (2.20 – 5.01)	<0.001	3.35 (2.23 – 5.04)	<0.001	3.33 (2.20 – 5.02)	<0.001
Haemoglobin, g/dL						
≥12 (F) or ≥13 (M)	1.00 (Reference)		1.00 (Reference)		1.00 (Reference)	
<12 (F) or <13 (M)	2.96 (2.07 – 4.23)	<0.001	2.94 (2.07 – 4.18)	<0.001	2.93 (2.05 – 4.18)	<0.001
Uric acid, mg/dL						
≤7.5	1.00 (Reference)		1.00 (Reference)		1.00 (Reference)	
>7.5	9.00 (5.82 – 13.92)	<0.001	8.99 (5.80 – 13.93)	<0.001	8.99 (5.81 – 13.92)	<0.001
C statistic (95% CI)	0.87 (0.85 – 0.90)		0.88 (0.86 – 0.90)		0.87 (0.85 – 0.90)	

†GFR ≥120 mL/min/1.73m^2

Abbreviations: CI, confidence interval; CKD, chronic kidney disease; CKD-EPI, the Chronic Kidney Disease Epidemiology Collaboration; F, female; eGFR, glomerular filtration rate; M, male; MDRD, the modification of diet in renal disease; OR, Odds ratio; T2DM, type 2 diabetes mellitus.

Figure S1. Study Flow on the Selection of Eligible Patients

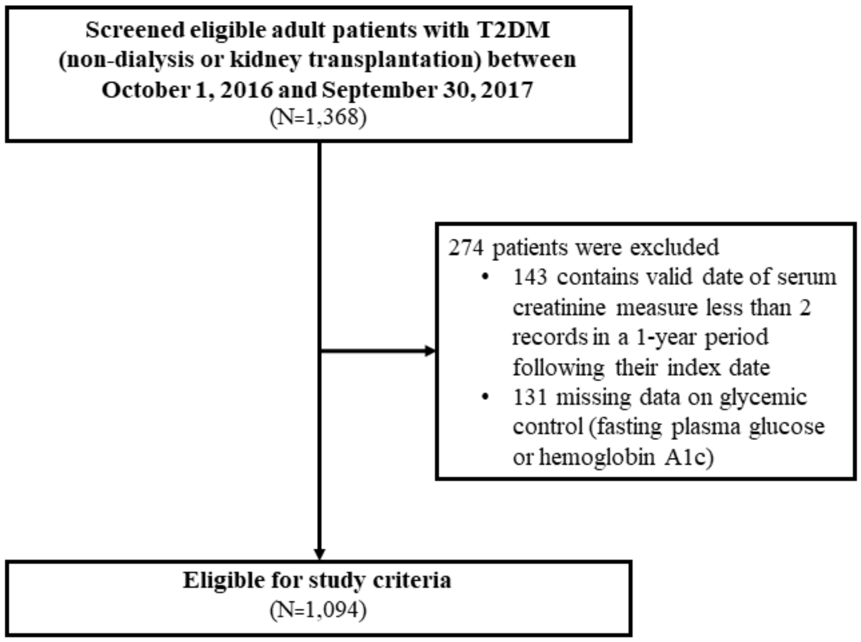

Abbreviations: T2DM, type 2 diabetes mellitus.

Figure S2. Age, Sex and Glycaemic Control Adjusted Prevalence Rates of CKD in Patients with T2DM According to Equation for Estimating GFR Expressed

A: CKD-EPI Equation for Asian

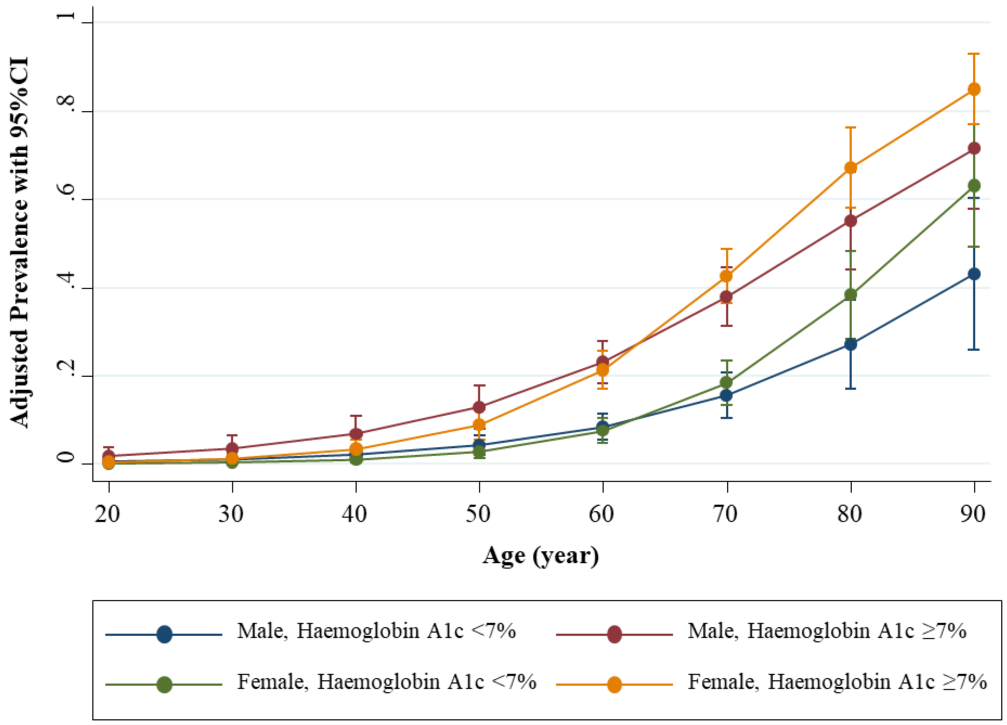

B: MDRD Equation

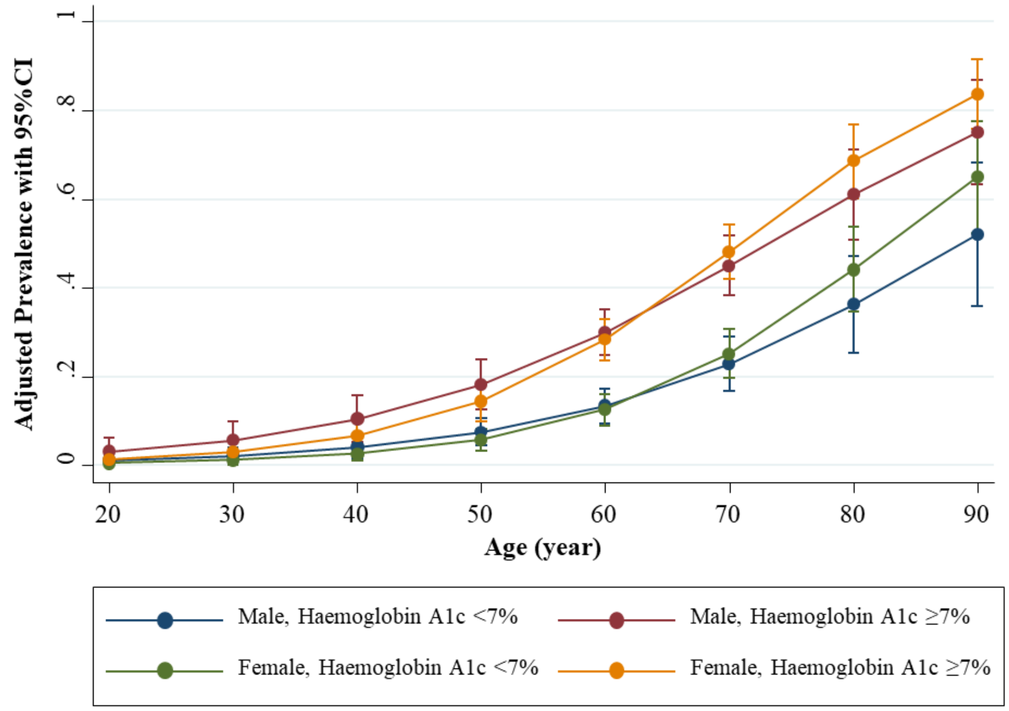

Abbreviations: CI, confidence interval; CKD, chronic kidney disease; CKD-EPI, the Chronic Kidney Disease Epidemiology Collaboration; eGFR, glomerular filtration rate; MDRD, the modification of diet in renal disease; T2DM, type 2 diabetes mellitus.

Figure S2. Age, Sex and Glycaemic Control Adjusted Prevalence Rates of CKD in Patients with T2DM According to Equation for Estimating GFR Expressed (Continued)

C: Thai GFR Equation

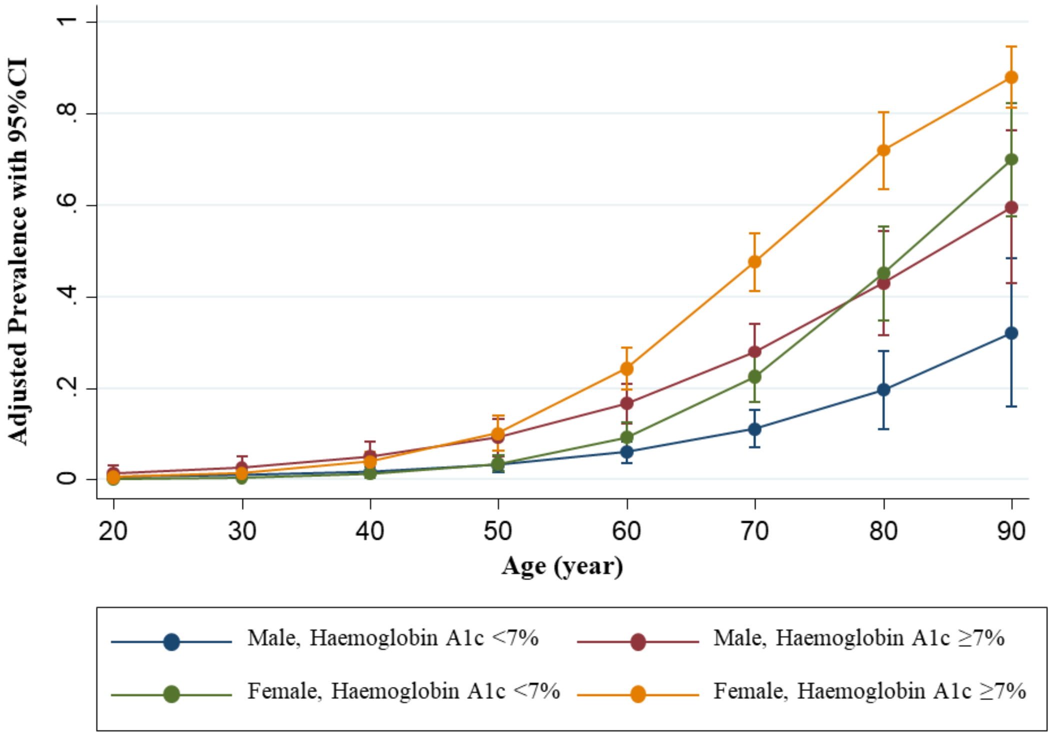

Abbreviations: CI, confidence interval; CKD, chronic kidney disease; CKD-EPI, the Chronic Kidney Disease Epidemiology Collaboration; eGFR, glomerular filtration rate; MDRD, the modification of diet in renal disease; T2DM, type 2 diabetes mellitus.

eReferences

1. Levey AS, Stevens LA, Schmid CH, Zhang YL, Castro AF 3rd, Feldman HI, et al; CKD-EPI (Chronic Kidney Disease Epidemiology Collaboration). A new equation to estimate glomerular filtration rate. Ann Intern Med. 2009;150(9):604-12.
2. Stevens LA, Claybon MA, Schmid CH, Chen J, Horio M, Imai E, et al. Evaluation of the Chronic Kidney Disease Epidemiology Collaboration equation for estimating the glomerular filtration rate in multiple ethnicities. Kidney Int. 2011;79(5):555-62.
3. Stevens LA, Coresh J, Feldman HI, Greene T, Lash JP, Nelson RG, et al. Evaluation of the modification of diet in renal disease study equation in a large diverse population. J Am Soc Nephrol. 2007;18(10):2749-57.
4. Praditpornsilpa K, Townamchai N, Chaiwatanarat T, Tiranathanagul K, Katawatin P, Susantitaphong P, et al. The need for robust validation for MDRD-based glomerular filtration rate estimation in various CKD populations. Nephrol Dial Transplant. 2011;26(9):2780-5.

Appendix. STROBE Statement—Checklist of items that Should be included in reports of ***cross-sectional studies***

	Item No	Recommendation	Page#
**Title and abstract**	1	*(a)* Indicate the study's design with a commonly used term in the title or the abstract	1
		(b) Provide in the abstract on informative and balanced summary of what was done and what was found	3
**Introduction**			
Background/ rational	2	Explain the scientific background and rationale for the investigation being reported	4, 5
Objectives	3	State specific objectives, including any prespecified hypotheses	5
**Methods**			
Study design	4	Present key elements of study design early in the paper	13
Setting	5	Describe the setting, locations, and relevant dates, including periods of recruitment, exposure, follow-up, and data collection	13
Participants	6	*(a)* Give the eligibility criteria, and the sources and methods of selection of participants	14
Variables	7	Clearly define all outcomes, exposures, predictors, potential confounders, and effect modifiers. Give diagnostic criteria, if applicable	14, 15
Data sources/ measurement	8*	For each variable of interest, give sources of data and details of methods of assessment (measurement). Describe comparability of assessment methods if there is more than one group	13
Bias	9	Describe any efforts to address potential sources of bias	14, 15
Study size	10	Explain how the study size was arrived at	15
Quantitative variables	11	Explain how quantitative variables were handled in the analyses. If applicable, describe which groupings were chosen and why	15, 16
Statistical methods	12	(a) Describe all statistical methods, including those used to control for confounding	15, 16
		(b) Describe any methods used to examine subgroups and interactions	NA
		(c) Explain how missing data were addressed	16
		(d) if applicable, describe analytical methods taking account of sampling strategy	NA
		(e) Describe any sensitivity analyses	16, 17
**Results**			
Participants	13*	*(a)* Report numbers of individuals at each stage of study—eg numbers potentially eligible, examined for eligibility, confirmed eligible, included in the study, completing follow-up, and analyzed	6
		(b) Indicate number of participants with missing data for each variable of interest	6, Table: 1
		(c) Consider use of a flow diagram	Figure S1

Appendix. STROBE Statement—Checklist of items that Should be included in reports of ***cross-sectional studies*** (Continued)

	Item No	Recommendation	Page#
**Results**			
Descriptive data	14*	*(a)* Give characteristics of study participants (eg demographic, clinical, social) and information on exposures and potential confounders	6
		(b) Indicate number of participants with missing data for each variable of interest	6, Table: 1
Outcome data	15*	Report numbers of outcome events or summary measures	6, 7 Table: 2-3
Main results	16	*(a)* Give unadjusted estimates and, if applicable, confounder-adjusted estimates and their precision (eg 95% confidence interval). Make clear which confounders were adjusted for and why they were included	6, 7 Table: 2-3
		**(b)** Report category boundaries when continuous variables were categorized	6, 7 Table: 2-3
		(c) If relevant, consider translating estimates of relative risk into absolute risk for a meaningful time period	7
Other analyses	17	Report other analyses done—eg analyses of subgroups and interactions, and sensitivity analyses	8, Table S3-S6, Figure S2
**Discussion**			
Key results	18	Summarize key results with reference to study objectives	8
Limitations	19	Discussion limitations of the study, taking into account sources of potential bias or imprecision. Discussion both direction and magnitude of any potential bias	11, 12
Interpretation	20	Give a cautions overall interpretation of results considering objectives, limitations, multiplicity of analyses, results from similar studies, and other relevant evidence	8-11
Generalizability	21	Discuss the generalizability (external validity) of the study results	11, 12
**Other information**			
Funding	22	Give the source of funding and the role of the funders for the present study and, if applicable, for the original study on which the present article is based	24

*Give information separately for exposed and unexposed groups
